# Genome-wide association study provides strong evidence of genes affecting the reproductive performance of Nellore beef cows

**DOI:** 10.1371/journal.pone.0178551

**Published:** 2017-05-31

**Authors:** Thaise Pinto de Melo, Gregório Miguel Ferreira de Camargo, Lucia Galvão de Albuquerque, Roberto Carvalheiro

**Affiliations:** 1 Department of Animal Science, School of Agricultural and Veterinarian Sciences, FCAV/ UNESP – Sao Paulo State University, Jaboticabal, Sao Paulo, Brazil; 2 Department of Animal Science, School of Veterinary Medicine and Animal Science, Federal University of Bahia, Salvador, Bahia, Brazil; 3 National Council for Scientific and Technological Development (CNPq), Brasília, DF, Brazil; China Agricultural University, CHINA

## Abstract

Reproductive traits are economically important for beef cattle production; however, these traits are still a bottleneck in indicine cattle since these animals typically reach puberty at older ages when compared to taurine breeds. In addition, reproductive traits are complex phenotypes, i.e., they are controlled by both the environment and many small-effect genes involved in different pathways. In this study, we conducted genome-wide association study (GWAS) and functional analyses to identify important genes and pathways associated with heifer rebreeding (HR) and with the number of calvings at 53 months of age (NC53) in Nellore cows. A total of 142,878 and 244,311 phenotypes for HR and NC53, respectively, and 2,925 animals genotyped with the Illumina Bovine HD panel (Illumina^®^, San Diego, CA, USA) were used in GWAS applying the weighted single-step GBLUP (WssGBLUP) method. Several genes associated with reproductive events were detected in the 20 most important 1Mb windows for both traits. Significant pathways for HR and NC53 were associated with lipid metabolism and immune processes, respectively. MHC class II genes, detected on chromosome 23 (window 25-26Mb) for NC53, were significantly associated with pregnancy success of Nellore cows. These genes have been proved previously to be associated with reproductive traits such as mate choice in other breeds and species. Our results suggest that genes associated with the reproductive traits HR and NC53 may be involved in embryo development in mammalian species. Furthermore, some genes associated with mate choice may affect pregnancy success in Nellore cattle.

## Introduction

Heifer rebreeding (HR) is the ability of a primiparous cow to rebreed after having the first calving—is an important trait in beef cattle production. Heifers that conceive soon after the first calving permit a fast return on investments. HR has been considered as a selection criterion in some breeding programs, particularly in indicine breeds [1, 2] as cows of these breeds usually present low rebreeding rates [3]. This trait shows a high genetic correlation with other important reproductive traits such as age at first calving [4] and stayability [5].

An alternative selection criterion for HR is the number of calving at 53 mo (NC53) [6]. In addition to discriminating between cows with 2 calves and those that did not rebreed as primiparous (1 calf), NC53 also allows considering in the genetic evaluation heifers that did not get pregnant. Compared with stayability, NC53 allows more emphasis on rebreeding of primiparous cows, one of the main bottlenecks for improving efficiency of beef cows in the tropics, besides allowing anticipating the genetic evaluation of sires using information of their progeny.

Despite the economic importance of HR and NC53, the genetic improvement of these traits is challenging because they present low heritability [5, 6], are expressed relatively late in life and just in females, which present lower selection intensity compared to males. As a consequence, genetic evaluations for these traits using only pedigree and phenotypic information result in expected breeding values with low accuracy. Trying to overcome this, genome-wide association studies (GWAS) have been performed with the hope to detect genomic regions associated to reproductive traits, aiming to use this information as a complementary tool to increase genetic gain.

In general, GWAS of reproductive traits have confirmed the polygenic nature of these traits. Nevertheless, some studies have identified important candidate genes for reproductive traits of beef cattle [7, 8, 9, 10, 11]. Conducting GWAS in independent populations is important to reinforce the association of some genomic regions with the trait of interest. When a genomic region is detected as important by different studies the evidence that this region harbor a true QTL is supposedly increased. Carrying out function analyses with the candidate genes may also help to unravel pathways and key genes involved in the expression of the trait of interest. In this study, we conducted GWAS and functional analyses aiming to identify important candidate genes and pathways associated with HR and NC53 in *Bos indicus* Nellore cows.

## Material and methods

### Phenotypes

Phenotypic information of Nellore cows was obtained from the Aliança Nelore database. The cows were born from 1984 to 2010 and were raised on 188 different commercial farms located in the southeastern, western and central regions of Brazil and in Paraguay. The feeding system adopted by these farms basically consists of tropical pastures, mineral salt and water *ad libitum*. In the dry season, the cows usually receive mineral supplementation.

During the breeding season, which lasts about 90 days and usually occurs in the rainy period, the heifers are either artificially inseminated or naturally mated. Generally, the first mating of heifers occurs at about 26 months of age, although some herds expose the heifers earlier at around 14–18 months of age in an anticipated mating season. In the data analyzed, 46.6% (66,643) of the heifers were exposed early, corresponding to an early pregnancy rate of 21.4% (14,287). Heifers that were exposed early and did not conceive had a second chance at about 26 months of age in the regular mating season, and all heifers that did not get pregnant during this period were culled, including those that were exposed for the first time. During the breeding season, the cows were either artificially inseminated or naturally mated using single or multiple sires (4–5 sires). There were 48.08%, 47.58% and 4.35% of calves born from artificial insemination, multiple sires and single sires, respectively.

Heifer rebreeding was defined as success (1) or failure (0), i.e., heifers that calved or not, respectively, since they had produced the first calf. NC53 was defined as 0, 1 or 2 for those heifers that did not have any calf, one calf or two calves at 53 months of age, given they had the opportunity to reach this age and had performance records until long-yearling age [6].

During editing, heifers with age at first calving less than 21 or greater than 40 months, age at second calving less than 32 or greater than 53 months, and a calving interval less than 11 months were excluded. The remaining number of phenotypes after editing was 142,878 and 244,311 for HR and NC53, respectively. For HR, 45.1% of the heifers failed and 54.9% succeeded to rebreed. For NC53, 38.0%, 28.6% and 33.4% had 0, 1 or 2 calves, respectively. The pedigree file for the two traits contained 369,878 animals distributed over five generations.

### Genotypes

A total of 2,925 animals were genotyped with the Illumina Bovine HD panel (Illumina^®^, San Diego, CA, USA). These animals included 2,212 Nellore heifers/cows from 12 different herds and 713 Nellore sires that had an average of 73.6 offspring evaluated for HR and NC53. The genotyped heifers and sires were born from 2002 to 2009 and from 1965 to 2006, respectively.

Quality control of genotypes was performed excluding single nucleotide polymorphisms (SNPs) from non-autosomal regions, SNPs mapped to the same position, and SNPs with a p-value for Hardy-Weinberg equilibrium < 10^−5^, a GC score < 0.15, a call rate < 0.95 and a minor allele frequency < 0.02. Samples with a call rate < 0.9 were discarded. The remaining number of SNPs and then samples after quality control was 409,376 and 2,923, respectively.

### Statistical analysis

The SNP marker effects were estimated using the weighted single-step GBLUP (WssGBLUP) method proposed by Wang et al. [12]. This method was chosen because it permits to combine pedigree, phenotypic and genomic information in a single step, weighting the SNP marker effects according to the variance explained by each SNP, i.e., a higher weight is attributed to the SNP that explains a higher proportion of the genetic variance. This method is particularly appealing when there are many more phenotypes than genotypes as in the present study. Phenotypic information from non-genotyped animals allows predicting more accurately the genetic merit of genotyped animals (*a*_*g*_). As the SNP effect estimates from WssGBLUP are computed as a function of *a*_*g*_, better predictions of *a*_*g*_ would ultimately result in better estimation of SNP effects [13]. The WssGBLUP analyses were run using the BLUPF90 family programs [14]. The method first computes the breeding values then the SNP effects, as described below.

Predicted breeding values were obtained with the following threshold animal model [15]:

$$y\text{=}X\beta \text{+}Z_{a}a\text{+}e\text{,}$$

where *y* is a vector of underlying liabilities for HR and NC53, *β* is a vector of fixed effects of the contemporary group, *a* is a vector of random additive direct genetic effects (breeding values), *X* and *Z*_*a*_ are incidence matrices relating elements in *β* and *a* to *y*, respectively, and *e* is the vector of random residuals. Contemporary groups were defined by concatenating the information of herd, year and season of birth, and weaning and yearling management groups of the heifers. Contemporary groups containing fewer than five heifers and without variability in HR or NC53, i.e., groups consisting of animals with the same categorical response, were excluded from the data. The underlying liabilities for HR and NC53 were defined as follows: HR = 0, if y < t1; HR = 1, if y > t1; NC53 = 0, if y < t1; NC53 = 1, if t1 < y < t2, and NC53 = 2, if y > t2, where t1 and t2 are the thresholds corresponding to the discontinuity in the observed scale of HR and NC53.

The covariance between *a* and *e* was assumed to be absent and their variances were equal to $H\sigma_{a}^{2}$ and $I\sigma_{e}^{2}$, respectively, where $\sigma_{a}^{2}$ and $\sigma_{e}^{2}$ are the additive direct and residual variances, respectively, *H* is the matrix which combines pedigree and genomic information [16], and *I* is an identity matrix. Since the variable in the underlying distribution is not observable, the parameterization $\sigma_{e}^{2}=1$ was adopted [17].

The parameter estimates of the threshold model were obtained under a Bayesian framework using the THRGIBBS1F90 Gibbs sampling program [18]. Default prior distributions were assumed for the variance components and for the fixed and random effects. The Gibbs sampler was run in a single chain of 500,000 iterations, with a burn-in period of 50,000 and a thinning interval of 50 iterations, totaling 9,000 posterior samples for each parameter to be estimated. The posterior means of the samples were used as the parameter estimates. The convergence of Monte Carlo chains of $\sigma_{a}^{2}$ and heritability was evaluated using the postGibbsf90 software [14] and the Geweke test of the BOA R package [19].

After computing the breeding values, the solutions of SNP effects (*û*) were then obtained according to VanRaden et al. [20] and Stranden & Garrick [21]: *û* = *DZ*′*[ZDZ*′*]*^−*1*^ *â*_*g*_, where *D* is a diagonal matrix with weights for SNP effects, *Z* is a matrix relating genotypes of each locus, and *â*_*g*_ is the vector of predicted breeding values of genotyped animals. The SNP effects breeding values, the *D* matrix and the SNP effects were iteratively recomputed over two iterations, as suggested by Wang et al. [12]. This number of iterations was chosen based on the results of a simulation study [13].

The diagonal elements of *D* (*d*_i_) were calculated as: *d*_*i*_ = *û*_*i*_^*2*^*p*_*i*_*(1-p*_*i*_*)*, where *û*_*i*_ is the allele substitution effect of the *i*^*th*^ marker estimated from the previous iteration, and *p*_*i*_ is the allele frequency of the second allele of the *i*^*th*^ marker [12]. Prior to recomputing *û*, the *D* matrix was normalized to enforce the total genetic variance to be constant across iterations.

### Detection of significant windows and functional groups

The proportion of variance explained by SNPs within non-overlapping consecutive 1-Mb windows was evaluated for both traits. A total of 2,523 windows spanning all autosomes were considered, with an average density of 162±48 SNPs per window. S1 and S2 Tables show the top 20 significant windows for HR and NC53, respectively. Annotated genes located within these windows were further inspected. The list of genes was provided by the NCBI Map Viewer tool (www.ncbi.nlm.nih.gov/mapview/) using the *Bos taurus* Annotation Release 103 and *Bos taurus* UMD 3.1 as reference assembly.

The DAVID software [22, 23], ClueGO program [24] and Cytoscape plug-in [25] were used to group genes according to similarity of the biological processes in which they are involved, aiming to verify if these functional clusters were related to reproductive events.

## Results and discussion

### Heritability estimates

The Geweke test [26] indicated convergence of the chains (Table 1). The heritability was 0.19 for HR and NC53 (Table 1), in agreement with the estimates reported in other studies on the Nellore breed [2, 5, 6]. This result indicates that, in addition to being highly influenced by the environment, HR and NC53 are affected by an additive genetic component, a finding justifying the execution of GWAS.

**Table 1:** Estimates of marginal posterior distributions of heritability for heifer rebreeding (HR) and number of calves at 53 months of age (NC53) of Nelore cattle.

	HR	NC53
Heritability^a^	0.194 ± 0.013	0.185 ± 0.008
HPD 95%	0.168–0.218	0.171–0.200
z-score^b^	-0.576	-0.989
p-value^b^	0.565	0.323
ESS	149.3	388.1

HPD 95%, 95% highest posterior density interval (inferior—superior); ESS, Effective Sample Size.

^a^Mean±s.d.

^b^Statistics of Geweke Test.

### Significant windows

S1 and S2 Tables show the BTA and position of the top 20 1-Mb significant windows for HR and NC53, respectively, as well as the percentage of genetic additive variance explained by each window and the genes within in each window. All significant genes are described below.

In S1 Table, window 71-72Mb of BTA11 harbors the *FOSL2* gene related to endometrial decidualization [27], *RBKS* associated with oocyte maturation [28], and *BRE* associated with steroidogenesis in the ovary and maintenance of placental function [29]. The subsequent window of BTA11 (72-73Mb) harbors important genes previously reported to be associated with reproductive events, such as *SLC30A3* [30] and *CAD* [31] described as estrogen receptors, *CIB4* associated with male sheep fecundity [32], *EMILIN1* associated with placentation and trophoblast invasion in the uterine wall [33], *TCF23* which plays an important role in endometrial decidualization [34], *UCN* associated with steroidogenesis and maintenance of placental function which is expressed in mature mouse spermatozoa and rat epididymis [35, 36, 37], and *IFT172*, *PREB* and *SNX17* which are associated with neural development [38, 39, 40, 41]. Furthermore, window 49-50Mb of BTA17 has been reported to be associated with age at first calving in Nellore cows [13].

As shown in S2 Table, window 6-7Mb of BTA3 harbors the *DDR2* gene, which has been associated with first calving and HR in Nellore cattle [10] in a study that used a subset of the present dataset. Window 39-40Mb of BTA9 harbors the *AMD1* gene, which is overexpressed in bovine oocyte and cumulus cells [42]. In addition, the AMD1 protein was located in luminal and epithelial cells of bovine endometrium [43]. This window also harbors *FYN* associated with mouse oocyte maturation [44] and maternal-fetal immune tolerance in humans and mice [45], *REV3L* which contributes to genome stability during neoplastic transformation and progression in mice—targeted disruption of this gene results in lethality during midgestation [46, 47], and *SLC16A10* which is differentially expressed in human placenta at mid-gestation, regulating embryonic development and growth [48]. Window 70-71Mb of BTA 17 was significant for both traits. This window harbors *CHEK2* which is down-regulated in human oocytes cryopreserved by slow freezing, reducing oocyte development competence [49], and plays an important role in DNA damage repair in mouse oocyte meiosis [50], and *XBP1*, a regulator gene associated with endoplasmic reticulum stress, influencing oocyte maturation and embryo development [51]. In pigs, *XBP1* expression promoted oocyte maturation, embryonic genome activation and early embryonic development *in vitro* [52]. In cattle, *XBP1* is involved in corpus luteum development and maintenance [53], and is upregulated in large follicles [54]. Window 18-19Mb of BTA26 harbors *FRAT1* which is differentially expressed during the secretory phases of the human endometrium [55], and *PGAM1* related to spermatogenesis success in mice [56] whose expression was found to be increased in cows with postpartum infection caused by pathogenic bacteria [57].

Some genes (*SLIT1*, *TNR*, *FAM181B*, *IFT172*, *PPM1G*, *SNX17*, and *PREB*) associated with neural development [38, 39, 40, 41, 58, 59, 60, 61, 62, 63] were also found in the top 20 significant windows for both traits. Costa et al. [10] also detected genes related to neural development that were associated with reproductive traits. Neurulation is an important and one of the first events of embryogenesis [64]. Failure in this stage can result in a non-viable pregnancy, a fact that would explain this association. Genes associated with male fertility [32, 36, 37, 56] were also found within the top 20 windows (*CIB4*, *UCN*, and *PGAM1*), a finding that would explain the genetic correlation between male and female reproductive traits as reported by Irano et al. [11]. Another possible explanation is that sperm quality influences pregnancy success. Thus, these genes may be associated with andrological parameters that also improve conception rates [65].

### Functional gene grouping

The top GO terms for the five functional groups with the highest enrichment score for HR and NC53 obtained by DAVID analyses are shown in Tables 2 and 3, respectively. The genes associated with HR were generally distributed in pathways related to metabolism and molecular functions. The top GO term for HR was “intracellular organelle lumen”. Functional group 2 (fatty acid metabolism) contained genes that play a role in the catabolism of short- (*ACADS* on BTA17 at 65-66Mb) and long-chain (*HADHA* and *HADHB* on BTA11 at 73-74Mb) fatty acids. The supply of lipophilic compounds such as fatty acids and some vitamins across the placenta influences growth and fetal fat mass formation [66]. Moreover, fatty acids are precursors of many sex hormones [67] and may influence the estrous cycle and pregnancy maintenance.

**Table 2:** Gene category and pathway enrichment analysis of HR genes.

Functional groups	Top Go Term (Id~Name)	Ontology	ES	FDR
1	GO: 0070013≈ Intracellular Organelle lumen	Cellular Component	1.17	5.4E1
2	Kegg: Fatty acid metabolism		1.08	2.0E1
3	Zinc finger region: RING-type		0.73	3.5E1
4	GO:0005509~Calcium ion binding	Molecular Function	0.60	9.4E1
5	Microtubule		0.55	8.9E1

ES, Enrichment Score; FDR, False Discovery Rate.

**Table 3:** Gene category and pathway enrichment analysis of NC53 genes.

Functional groups	Top Go Term (Id~Name)	Ontology	ES	FDR
1	Go:0042613~MHC class II Protein complex	Cellular Component	7.81	7.3E-2
2	Kegg: Asthma		5.10	1.8E-4
3	GO: 0000166~Nucleotide binding	Molecular Function	0.93	7.0E1
4	GO: 0046872~Metal ion binding	Molecular Function	0.52	9.8E1
5	GO: 0006915~Apoptotic process	Biological Process	0.40	1.0E2

ES, Enrichment Score; FDR, False Discovery Rate.

Functional group 5 (microtubule) contained the *DYNLL1*, *KIF3C* and *TRIM54* genes (BTA17 at 65-66Mb, BTA11 at 73–74 Mb and BTA11 at 72-73Mb, respectively). The *DYNLL1* and *KIF3C* genes encode two motor proteins called kinesins and dyneins that regulate intracellular transport through microtubules. These genes are associated with the mitotic process and play important roles in embryo development and cell migration and differentiation [68, 69, 70, 71]. Microtubules are responsible for many intracellular movements, including chromosome separation during cell division. When a cell enters mitosis, the disassembly rate of microtubules increases about ten-fold and the number of microtubules increases by five- to ten-fold [72]. Since mitosis is also essential for embryonic growth and development [73], these microtubule genes affect the success of embryo development.

For NC53, most genes were associated with immune processes. The top GO term for this trait was “MHC class II protein complex”, which is directly associated with the immune system and pregnancy success [74]. This group of genes will be further discussed below.

**Fig 1:**
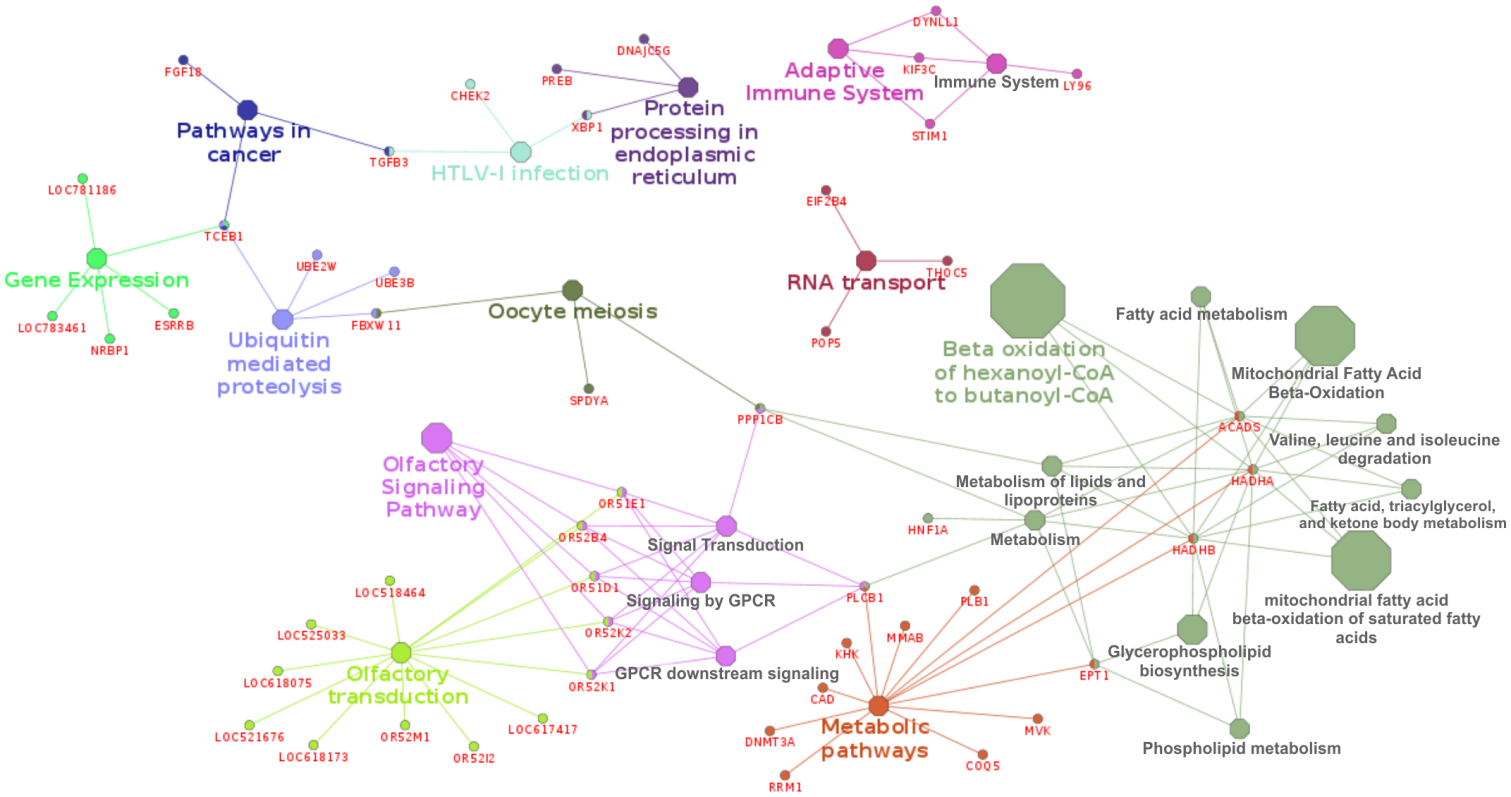
Gene network of heifer rebreeding. Each color represents a functional group that harbored related subgroups and genes.

The Cytoscape software was used to cluster the genes into functional groups. For HR, 25 GO terms/groups were generated (Fig 1) and three of them were statistically oversignificant (p < 0.001), namely “beta-oxidation of hexanoyl-CoA to butanoyl-CoA” (the most significant with p = 2.77E-6), “mitochondrial fatty acid beta-oxidation of saturated fatty acids”, and “mitochondrial fatty acid beta-oxidation”. These pathways are associated with lipid metabolism, which was a significant functional group in DAVID analysis. The genes grouped in these pathways were the same as those identified by DAVID analysis (*ACADS*, *HADHA*, and *HADHB*).

Metabolism of fatty acids is directly associated with the pregnancy success. Females with no satisfactory corporal condition that do not receive a good nutrition supplementation after calving have higher interval from calving to estrus, decrease conception rate and produce lighter calves [75]. The significant pathways related to lipid metabolism might suggest that there are cows that accumulate more fat in comparison to others. As a consequence, they have a better rebreeding performance.

The “Olfactory signaling pathways” was a significant cluster (0.05 < p-value < 0.01). Genes clustered in this group were: *OR51D1*, *OR51E1*, *OR52B4*, *OR52K1*, *OR52K2* (BTA15, at 51-52Mb). Two of these, *OR51D1* and *OR52K1* were found to be expressed in female and male primordial germ cells and in unfertilized human oocytes, participating in gamete production [76]. Furthermore, olfactory receptors integrate many metabolic processes and some of them play important role in reproduction [76].

“Oocyte meiosis” is also a pathway extremely related with reproductive events. Despite this pathway was not significant, it clustered three genes associated with HR: *FBXW11*, *PPP1CB*, *SPDYA*. The *PPP1CB* gene is also associated to “Olfactory” pathway. This gene plays a role in regulation of oocytes chromatin condensation in mouse [77].

**Fig 2:**
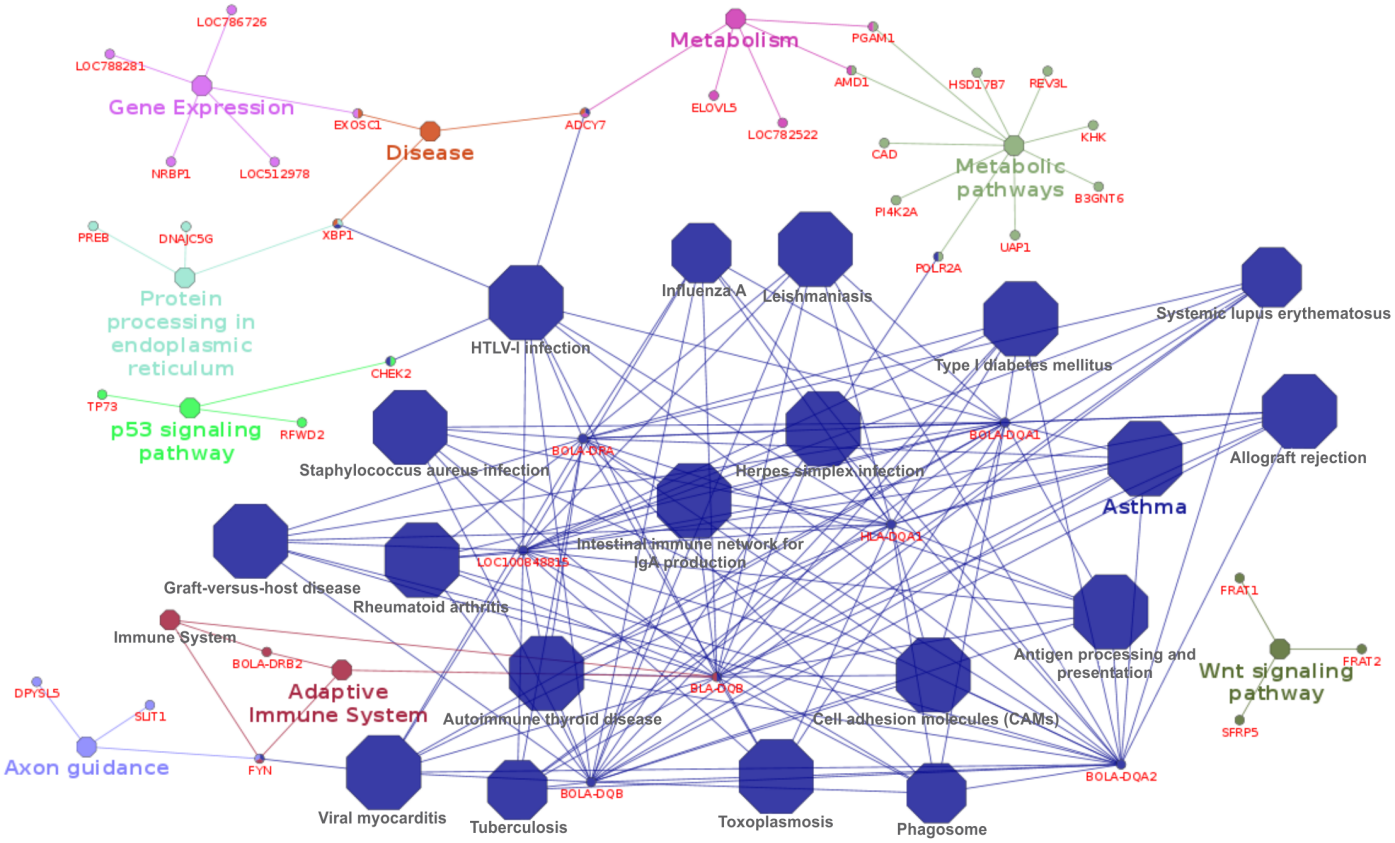
Gene network of number of calving at 53 months of age. Each color represents a functional group that harbored related subgroups and genes.

For NC53, 29 GO terms/groups were generated, 19 of them were over significant, that are presented in dark-blue in Fig 2. “Asthma” was the group that presented the higher significance level (p-value = 1.12E-9). The signalized genes from the disease pathways were the ones from the Major Histocompatibility Complex Class II (MHC II). Shortly, the MHC complex codifies cell surface glycoproteins that present the peptide antigen to the T-cells [78]. MHC class II molecules can stimulate the proliferation of some inflammatory cytokines contributing to chronic inflammation responses, as observed in some human autoimmune diseases [79,80]. It is indirectly related to the maternal-fetal tolerance and embryo development [81]. This association between MHC class II and autoimmune diseases could explain why human diseases pathways were found by Cytoscape analyses.

MHC is composed of a group of highly polymorphic genes and its variability is important to enable the immune system to recognize and act against pathogens [82]. The genes listed were *BLA-DQB*, *BOLA-DQA1*, *BOLA-DQA2*, *BOLA-DQB*, *BOLA-DRA*, and *HLA-DQA1*, located on BTA23 at 25-26Mb.

In addition to the important immune function of MHC, evidence indicates that males and females choose MHC-dissimilar sexual partners [83], which works as a self/nonself perception to guarantee that their offspring will be immunogenetically viable, consequently ensuring the survival of the species. The more polymorphic the MHC, the higher the probability of fighting efficiently different diseases. Aarnink et al. [74] observed a low survival rate in a Mauritian-origin macaque population with a low level of polymorphism in MHC genes. Negative natural selection acts against offspring whose parents have similar MHC alleles. In beef cattle reproduction, the preference of some bulls for certain cows in single/multiple sire mating might be explained by the action of MHC genes.

The choice of the sexual partner occurs before copulation and is guided by dissimilarity in the MHC genes of the partners that are able to recognize MHC variability based on body odor [83]. Moreover, MHC-based mate choice continues during sperm-oocyte interaction (chemotaxis) after copulation and is mediated by olfactory receptors present on the gametes [76]. The olfactory receptors of the gametes “reflect” the MHC variability of the oocyte/sperm and guide fecundation between the genetically most distant gametes.

The association of MHC genes and olfactory receptors with NC53 allows raising some hypotheses linked to cattle production. For example, artificial insemination does not allow mate choice, suggesting that a dam could be inseminated with semen from a sire with similar MHC alleles. In this case, selection for MHC variability would only be possible during fecundation (gamete level). If MHC genes are similar, fecundation may not occur. Thus, unsuccessful rebreeding may also be explained by the similarity of MHC genes between sexual partners. The detection of some olfactory receptor genes and olfactory pathways associated with HR supports this hypothesis.

Evidently, these hypotheses need to be confirmed, but some changes in cattle handling could be proposed until the influence of MHC has been established. The replacement of sire semen after pregnancy failure and the use of natural mating for part of the females might promote higher pregnancy rates since these practices tend to better respect the natural MHC selection process. MHC genes are not the only reason for pregnancy failure, but the present results suggest that they may influence pregnancy rates and therefore deserve further attention.

## Conclusion

Genes that play important roles in embryo development, lipid metabolism and sexual partner choice were associated with reproductive traits in Nellore cattle. Further studies may confirm the biological role of these genes behind the pregnancy success in mammals.

## Supporting information

S1 Table Genes harbored in the top20 1Mb windows for heifer rebreeding. (DOCX)

S2 Table Genes harbored in the top20 1Mb windows for number of calving at 53 months of age. (DOCX)
