# Supplementary material for: Genome-wide association study provides strong evidence of genes affecting the reproductive performance of Nellore beef cows
Source: PLoS One. 2017 May 31;12(5):e0178551. doi: 10.1371/journal.pone.0178551 (PMC5451131; doi:10.1371/journal.pone.0178551)
Supplement: S1 Table — (DOCX) [file pone.0178551.s001.docx]

**Genome-wide association study provides strong evidence of genes affecting the reproductive performance of Nellore beef cows.** Thaise Pinto de Melo, Gregório Miguel Ferreira de Camargo, Lucia Galvão de Albuquerque, Roberto Carvalheiro*. *Corresponding author: Department of Animal Science, School of Agricultural and Veterinarian Sciences, FCAV/ UNESP – Sao Paulo State University, Jaboticabal, Sao Paulo, Brazil; e-mail: rcar@fcav.unesp.br

**S1 Table. Genes harbored in the top20 windows for heifer rebreeding.**

| **BTA** | **Position (Mb)** | **%var** | **Genes** | **GenBank Accession n^o^** |
| --- | --- | --- | --- | --- |
| 4 | 6-7 | 0.34 | VWC2, LOC101904266, LOC781773 | AC_000161 GPC_000000173 |
| 6 | 78-79 | 0.38 | TRNAC-GCA, ADGRL3 | AC_000163 GPC_000000175 |
| 6 | 110-111 | 0.36 | SLC2A9, TRNAG-CCC, WDR1, ZNF518B, LOC100335675, CLNK, LOC101905921, TRNAA-UGC | AC_000163 GPC_000000175 |
| 10 | 88-89 | 0.35 | TTLL5, TGFB3, IFT43, GPATCH2L, ESRRB, LOC104973251, LOC101903493, LOC104973252 | AC_000167 GPC_000000179 |
| 11 | 70-71 | 0.38 | LOC104968430, LOC101905929, ALK, CLIP4, C11H2orf71, FAM179A, TRNAS-GGA, LOC104973418, WDR43, TRMT61B, SPDYA, PPP1CB | AC_000168 GPC_000000180 |
| 11 | 71-72 | 0.67 | PLB1, LOC104973420, LOC104973419, LOC104973421, FOSL2^a^, LOC104973422, BRE^a^, LOC101904887, LOC101904806, RBKS^a^, MRPL33, SLC4A1AP, LOC101905151 | AC_000168 GPC_000000180 |
| **11** | **72-73^b^** | 0.92 | SLC4A1AP, SUPT7L, GPN1, CCDC121, ZNF512, LOC100141098, GCKR, FNDC4, IFT172^a^, KRTCAP3, NRBP1, PPM1G^a^, ZNF513, SNX17^a^, EIF2B4, GTF3C2, MPV17, UCN^a^, TRIM54, DNAJC5G, SLC30A3^a^, CAD^a^, ATRAID, SLC5A6, TCF23^a^, PRR30, PREB^a^, ABHD1, CGREF1, KHK, EMILIN1^a^, OST4, AGBL5, TRNAA-AGC, TRNAY-GUA, TMEM214, LOC101906001, MAPRE3, DPYSL5, CENPA, SLC35F6, KCNK3, CIB4^a^, TRNAE-UUC, C11H2orf70, OTOF | AC_000168 GPC_000000180 |
| 11 | 73-74 | 0.40 | OTOF, DRC1, EPT1, MIR2284Y-1, GPR113, LOC101906131, HADHB, HADHA, GAREML, LOC104973423, RAB10, LOC104973424, TRNAC-ACA, KIF3C, LOC104973425, LOC783461, ASXL2, DTNB, TRNAC-GCA, LOC100847382, LOC104973427, DNMT3A, MIR1301, LOC104968513 | AC_000168 GPC_000000180 |
| 13 | 1-2 | 0.35 | PLCB1, MIR2285M-1, LOC104973687, LOC104973688 | AC_000170 GPC_000000182 |
| 14 | 39-40 | 0.48 | STAU2, LOC104974049, MIR2284L, UBE2W, TCEB1, TMEM70, LY96, LOC104974050, LOC101904383, TRNAE-UUC, LOC104974052, LOC104974051, LOC100337130, LOC781682, JPH1, LOC104974056, LOC104974055, LOC104974054, GDAP1, LOC104974053 | AC_000171 GPC_000000183 |
| 15 | 51-52 | 0.40 | LOC104974262, OR51E1, OR51D1, TRIM68, OR52I2, LOC618050, LOC510351, LOC784727, LOC784689, OR52K2, LOC617417, OR52K1, OR52M1, LOC618067, LOC521676, LOC618075, TRIM21, LOC518464, LOC785574, LOC100138885, OR52B4, LOC517056, LOC785740, LOC785109, LOC519105, LOC618213, LOC785773, LOC506452, LOC785853, LOC100299115, LOC100298880, LOC782157, LOC784088, LOC619140, LOC785808, LOC788363, LOC519104, LOC100299247, TRNAG-CCC, LOC525033, LOC618173, RRM1, LOC101904526, STIM1 | AC_000172 GPC_000000184 |
| **16** | **50-51^b^** | 0.44 | C16H1orf174, DFFB, CEP104, MIR2320, LOC101906943, LOC104974456, LRRC47, SMIM1, CCDC27, TP73, WRAP73, TPRG1L, MEGF6, MIR551A, ARHGEF16 | AC_000173 GPC_000000185 |
| 17 | 49-50 | 1.04 | TMEM132D, LOC101907645, GLT1D1, LOC100140533, TRNAE-UUC, SLC15A4, TMEM132C, LOC101907809, LOC104974623 | AC_000174 GPC_000000186 |
| 17 | 65-66 | 0.38 | TRIAP1, GATC, SRSF9, DYNLL1, COQ5, RNF10, POP5, CABP1, MLEC, UNC119B, ACADS, LOC104974683, SPPL3, LOC104974672, HNF1A, LOC104974673, C17H12orf43, OASL, LOC101905682, LOC100296211, ANKRD13A, GIT2, TCHP, GLTP, TRPV4, FAM222A, LOC101903333, LOC104974674, LOC104974675, MVK, MMAB, UBE3B, KCTD10, MYO1H | AC_000174 GPC_000000186 |
| **17** | **70-71^c^** | 0.34 | TTC28, LOC101902902, TRNAE-CUC, CHEK2^a^, HSCB, LOC104974694, CCDC117, XBP1^a^, TRNAG-CCC, LOC101903066, LOC104970055, ZNRF3, LOC615587, KREMEN1, LOC104974695, EMID1, RHBDD3, EWSR1, GAS2L1, RASL10A, AP1B1, LOC100847257, NEFH, THOC5, NIPSNAP1 | AC_000174 GPC_000000186 |
| 20 | 3-4 | 0.43 | LOC533085, TLX3, TRNAK-UUU, NPM1, FGF18, LOC781186, LOC104975182, LOC104975183, SMIM23, LOC533234, FBXW11, LOC101902681, LOC101903110, STK10, LOC101903680, LOC101903388, LOC100138705, EFCAB9, UBTD2 | AC_000177 GPC_000000189 |
| 20 | 14-15 | 0.53 | ADAMTS6, LOC104975223, LOC104975224, CWC27, LOC784354, LOC104975225, SREK1IP1, FAM159B, RGS7BP | AC_000177 GPC_000000189 |
| 21 | 42-43 | 0.38 | STRN3, AP4S1, HECTD1, LOC101908185, HEATR5A, DTD2, GPR33, NUBPL, ARHGAP5 | AC_000178 GPC_000000190 |
| 26 | 42-43 | 0.33 | ATE1, LOC101907255, LOC104970731, NSMCE4A, TACC2, MIR2396, BTBD16, LOC104970753, LOC104970752, PLEKHA1, HTRA1, LOC100849037, DMBT1, LOC100295703, SPADH2, SPADH1, C26H10orf120, LOC100300910, LOC104968509, LOC617705, LOC104970756, TRNAG-CCC | AC_000183 GPC_000000195 |
| 29 | 13-14 | 1.14 | FAM181B^a^, LOC783542, TRNAC-GCA, LOC101903586, TRNAC-GCA, LOC104971240 | AC_000186 GPC_000000198 |

%var, Additive genetic variance proportion explained by the window.

^a^Genes associated with reproductive events.

^b^Windows in bold were in common between the traits heifer rebreeding and number of calvings at 53 months of age.
