# Supplementary material for: Genome-wide association study provides strong evidence of genes affecting the reproductive performance of Nellore beef cows
Source: PLoS One. 2017 May 31;12(5):e0178551. doi: 10.1371/journal.pone.0178551 (PMC5451131; doi:10.1371/journal.pone.0178551)
Supplement: S2 Table — (DOCX) [file pone.0178551.s002.docx]

**Genome-wide association study provides strong evidence of genes affecting the reproductive performance of Nellore beef cows.** Thaise Pinto de Melo, Gregório Miguel Ferreira de Camargo, Lucia Galvão de Albuquerque, Roberto Carvalheiro*. *Corresponding author: Department of Animal Science, School of Agricultural and Veterinarian Sciences, FCAV/ UNESP – Sao Paulo State University, Jaboticabal, Sao Paulo, Brazil; e-mail: rcar@fcav.unesp.br

**S2 Table. Genes harbored in the top20 windows for number of calving at 53 months of age.**

| **BTA** | **Position (Mb)** | **%var** | **Genes** | **GenBank Accession n^o^** |
| --- | --- | --- | --- | --- |
| 1 | 17-18 | 0.45 |  | AC_000158 GPC_000000170 |
| 3 | 6-7 | 0.55 | NUF2, LOC104970589, LOC104970606, RGS5, LOC104970616, RGS4, LOC104970648, C3H1orf110, LOC101907096, HSD17B7, DDR2^a^, LOC104970668, UAP1, LOC782522, LOC104970688, UHMK1 | AC_000160 GPC_000000172 |
| 8 | 68-69 | 0.95 | LOC104969388, LOC788281, LOC786417, TRNAC-GCA, LOC101904250, LOC104969389, LOC786270 | AC_000165 GPC_000000177 |
| 9 | 39-40 | 0.87 | LOC104969533, FYN^a^, LOC101905061, LOC104969535, TRAF3IP2, LOC784866, LOC616830, REV3L^a^, MIR6536-2, MIR6536-1, LOC104969536, KIAA1919, SLC16A10^a^, RPF2, LOC512978, GTF3C6, CDK19, AMD1^a^ | AC_000166 GPC_000000178 |
| 10 | 101-102 | 0.45 | KCNK10, SPATA7, PTPN21, ZC3H14, EML5, TTC8, LOC101907140, FOXN3, LOC101902748 | AC_000167 GPC_000000179 |
| **11** | **72-73^b^** | 0.69 | SLC4A1AP, SUPT7L, GPN1, CCDC121, ZNF512, LOC100141098, GCKR, FNDC4, IFT172^a^, KRTCAP3, NRBP1, PPM1G^a^, ZNF513, SNX17^a^, EIF2B4, GTF3C2, MPV17, UCN^a^, TRIM54, DNAJC5G, SLC30A3^a^, CAD^a^, ATRAID, SLC5A6, TCF23^a^, PRR30, PREB^a^, ABHD1, CGREF1, KHK, EMILIN1^a^, OST4, AGBL5, TRNAA-AGC, TRNAY-GUA, TMEM214, LOC101906001, MAPRE3, DPYSL5, CENPA, SLC35F6, KCNK3, CIB4^a^, TRNAE-UUC, C11H2orf70, OTOF | AC_000168 GPC_000000180 |
| 14 | 80-81 | 0.57 | RALYL, LOC104974140, LOC101904543, LOC616400 | AC_000171 GPC_000000183 |
| 15 | 36-37 | 0.50 | PLEKHA7, C15H11orf58, LOC101904740, SOX6 | AC_000172 GPC_000000184 |
| 15 | 57-58 | 0.63 | LOC104974276, ACER3, LOC786726, LOC104974277, B3GNT6, CAPN5, MYO7A, LOC786996, LOC786511, ANO3 | AC_000172 GPC_000000184 |
| **16** | **50-51^b^** | 0.49 | C16H1orf174, DFFB, CEP104, MIR2320, LOC101906943, LOC104974456, LRRC47, SMIM1, CCDC27, TP73, WRAP73, TPRG1L, MEGF6, MIR551A, ARHGEF16 | AC_000173 GPC_000000185 |
| 16 | 58-59 | 1.05 | TNR^a^, RFWD2, LOC101902053, LOC100848409 | AC_000173 GPC_000000185 |
| **17** | **70-71^b^** | 0.98 | TTC28, LOC101902902, TRNAE-CUC, CHEK2^a^, HSCB, LOC104974694, CCDC117, XBP1^a^, TRNAG-CCC, LOC101903066, LOC104970055, ZNRF3, LOC615587, KREMEN1, LOC104974695, EMID1, RHBDD3, EWSR1, GAS2L1, RASL10A, AP1B1, LOC100847257, NEFH, THOC5, NIPSNAP1 | AC_000174 GPC_000000186 |
| 18 | 17-18 | 0.57 | N4BP1, LOC104974765, LOC104974836, LOC101905800, LOC104974766, CBLN1, LOC104970029, LOC780820 | AC_000175 GPC_000000187 |
| 18 | 18-19 | 0.76 | ZNF423, TRNAG-CCC, LOC104974768, LOC104974767, LOC101906070, LOC786252, CNEP1R1, HEATR3, LOC104974769, PAPD5, ADCY7, BRD7 | AC_000175 GPC_000000187 |
| 23 | 25-26 | 0.55 | ICK, FBXO9, GCM1, LOC104975657, ELOVL5, BOLA-DQA2, BOLA-DQB, BOLA-DQA5, LOC100851058, LOC100848815, BOLA-DRB3, BOLA-DQA1, LOC786695, LA-DRB, BOLA-DRB2, LOC101902890, BOLA-DRA, BTNL2, LOC525599, LOC504295, LOC101903024, LOC101903077, LOC104969893, LOC101903155, LOC104975658, BLA-DQB, LOC783151, LOC101903211, LOC100337245 | AC_000180 GPC_000000192 |
| 24 | 18-19 | 0.71 | LOC505339, TRNAW-CCA | AC_000181 GPC_000000193 |
| 26 | 18-19 | 0.84 | LOC100849063, LOC100851323, SLIT1^a^, ARHGAP19, LOC104975958, LOC104975959, FRAT1^a^, FRAT2, RRP12, PGAM1^a^, EXOSC1, ZDHHC16, MMS19, UBTD1, ANKRD2, HOGA1, C26H10orf62, MORN4, PI4K2A, LOC104975960, AVPI1, MARVELD1, ZFYVE27, SFRP5, LOC104975961, GOLGA7B, LOC101905123, CRTAC1 | AC_000183 GPC_000000195 |
| 27 | 9-10 | 0.56 | LOC104976044 | AC_000184 GPC_000000196 |
| 28 | 14-15 | 0.53 | BICC1, TRNAG-CCC, LOC101906006, LOC104976173, POLR2A, LOC104976174, PHYHIPL, FAM13C | AC_000185 GPC_000000197 |
| 28 | 34-35 | 0.65 | LOC104976196, LOC104969726, LOC104969725, LOC104969724, LOC104971012, LOC104976197, LOC104971017, LOC104971019, LOC101907494, ZMIZ1, LOC104976196 | AC_000185 GPC_000000197 |

%var, Additive genetic variance proportion explained by the window.

^a^Genes associated with reproductive events.

^b^Windows in bold were in common between the traits heifer rebreeding and number of calvings at 53 months of age.
